# β2-spectrin depletion impairs DNA damage repair

**DOI:** 10.18632/oncotarget.9677

**Published:** 2016-05-27

**Authors:** Nobuo Horikoshi, Raj K. Pandita, Kalpana Mujoo, Shashank Hambarde, Dharmendra Sharma, Abid R. Mattoo, Sharmistha Chakraborty, Vijaya Charaka, Clayton R. Hunt, Tej K. Pandita

**Affiliations:** ^1^ Department of Radiation Oncology, Houston Methodist Research Institute, Houston, TX, USA; ^2^ Department of Radiation Oncology, University of Texas Southwestern Medical School, Dallas, TX, USA; ^3^ Department of Microbiology and Molecular Genetics, McGovern Medical School, University of Texas Health Science Center at Houston, Houston, TX, USA

**Keywords:** β2-spectrin, genotoxicity, DNA repair

## Abstract

β2-Spectrin (β2SP/SPTBN1, gene *SPTBN1*) is a key TGF-β/SMAD3/4 adaptor and transcriptional cofactor that regulates TGF-β signaling and can contribute to liver cancer development. Here we report that cells deficient in β2-Spectrin (β2SP) are moderately sensitive to ionizing radiation (IR) and extremely sensitive to agents that cause interstrand cross-links (ICLs) or replication stress. In response to treatment with IR or ICL agents (formaldehyde, cisplatin, camptothecin, mitomycin), β2SP deficient cells displayed a higher frequency of cells with delayed γ-H2AX removal and a higher frequency of residual chromosome aberrations. Following hydroxyurea (HU)-induced replication stress, β2SP-deficient cells displayed delayed disappearance of γ-H2AX foci along with defective repair factor recruitment (MRE11, CtIP, RAD51, RPA, and FANCD2) as well as defective restart of stalled replication forks. Repair factor recruitment is a prerequisite for initiation of DNA damage repair by the homologous recombination (HR) pathway, which was also defective in β2SP deficient cells. We propose that β2SP is required for maintaining genomic stability following replication fork stalling, whether induced by either ICL damage or replicative stress, by facilitating fork regression as well as DNA damage repair by homologous recombination.

## INTRODUCTION

Maintaining genomic stability is important for an organism throughout its lifetime in order to protect against the development of cancer. Alcohol-related genotoxicity, arising from DNA damage by metabolically generated reactive aldehydes, has recently been observed in animal models containing inactivation of genes in the Fanconi anemia pathway [1]. Specifically, *Fanc* mutant mice crossed with *Aldh2*-mutant mice are susceptible to ethanol-induced teratogenicity and display defective DNA interstrand cross-link repair [1]. Yet, alcohol-treated mice with the *Fanc* mutation alone do not develop fetal alcohol syndrome-like aberrations, suggesting the importance of genotoxic acetaldehyde in a complex process of toxin-induced DNA damage [2-5]. Transforming growth factor β (TGF-β) is a critical protein in the regulation of several cancer phenotypes [6] and keratinocytes of TGFβ1-null mice exhibit genomic instability [8]. TGF-β also functions as an extracellular sensor of IR-induced cell damage [7] and this function along with numerous other TGF-β actions are initiated through binding to the TGF-β receptor I and TGF-β receptor II serine-threonine kinase receptors, followed by activation, phosphorylation, and nuclear translocation of receptor regulated SMAD3 and SMAD2. The latter form complexes with the common mediator SMAD4 and subsequently activate TGF-β target genes [9]. Recent studies of Smad4 knockout mice, that develop head and neck cancers, demonstrated a significant role for *Smad4* in promoting genomic stability through regulation of the Fanconi anemia/BRCA DNA repair pathway [10]. Despite these observations, precisely how the TGF-β pathway contributes to toxin-induced DNA damage repair remains unclear.

Previous studies indicate that β2SP is a key TGF-β/Smad3/4 adaptor and transcriptional cofactor that can regulate TGF-β signaling and liver cancer development [11, 12]. β2-Spectrin is a dynamic intracellular non-pleckstrin homology (PH)-domain protein that belongs to a family of polypeptides implicated in cell polarity. Through associated binding partners, such as ankyrin, spectrins target and stabilize membrane proteins, such as ion transporters, exchangers, and cell adhesion molecules, in diverse tissues and cell types, including erythrocytes, gut, liver, and brain cells [13]. Spectrin dysfunction has previously been linked to abnormalities in mammalian physiology, including elliptocytosis, anemia, and cerebellar degeneration. More recently, spectrins have been linked to multiple signaling pathways, including cell cycle regulation, DNA repair, and TGF-β signaling [11, 14, 15]. *Sptbn1* heterozygous mice are robust genetic models of liver malignancies associated with loss of TGF-β signaling, with more than 40% of mice spontaneously developing liver tumors [9, 12, 16-18]. Homozygous loss of *Sptbn1* in mouse is embryonic lethal due to multiple abnormalities of the liver, gut, and brain, indicating an essential role in embryogenesis [11]. Here, we report that *Sptbn1*-null cells or cells deficient in β2SP display a marked defect in DNA damage repair by the homologous recombination (HR) pathway in response to various exogenous mutagens.

## RESULTS

Homozygous *Sptbn1* null (*Sptbn1*^−/−^ or *β2SP*^−/−^) mice are embryonic lethal. Mutant embryos examined at E11.5 post coitus (p.c.) display a loss of TGF-β signaling and a number of morphological abnormalities such as retarded growth, liver and gut hypoplasia, cardiac muscle hypertrophy, and neural defects [11]. Interestingly, a similar phenotype was described for double knockout *Aldh2*^−/−^*/Fancd2*^−/−^ mice in a study demonstrating that the acetaldehyde-catabolizing enzyme aldehyde dehydrogenase 2 (Aldh2) is essential for embryonic viability of mice deficient in the Fanconi anemia complementation group D2 (*Fancd2*) DNA repair gene [1]. Since *Aldh2*^−/−^*/Fancd2*^−/−^ mice are remarkably sensitive to ethanol exposure, both *in utero* and postnatally, we asked whether cells deficient for β2SP are sensitive to DNA damaging agents.

### β2SP depletion exhibits spontaneous genomic instability

**Figure 1:**
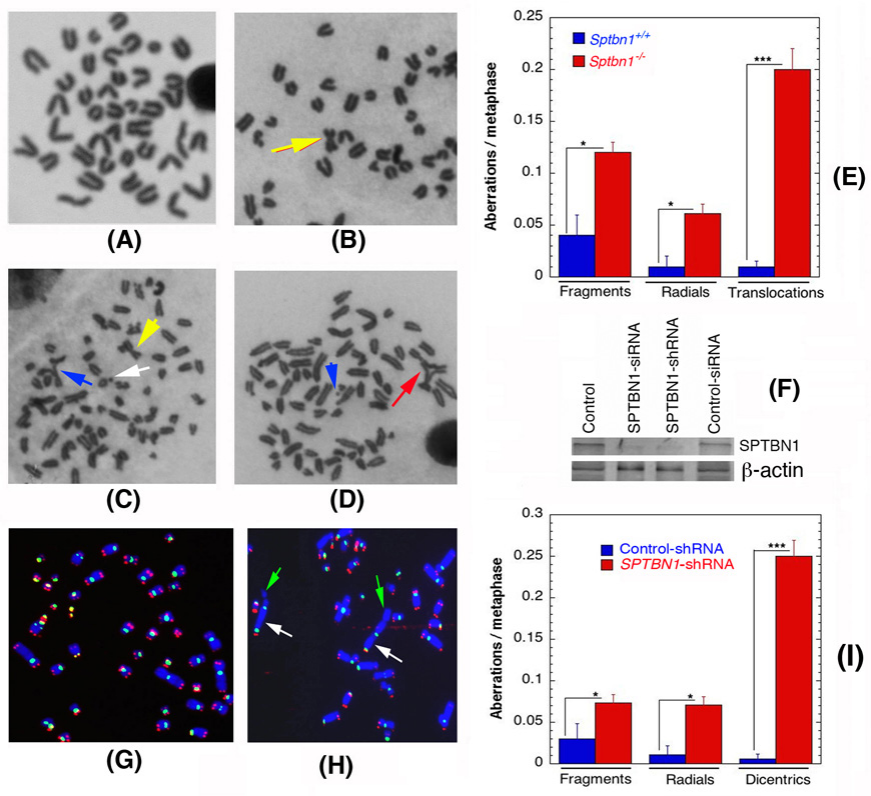
Spontaneous genomic instability after β2SP depletion in mouse embryonic fibroblasts (*Sptbn1*−/−) and human cells **A.**, **B.** Metaphases from wild type *Sptbn1*^+/+^ MEFs. A. Metaphase with normal telocentric chromosomes and **B.** metaphase with one metacentric chromosomes like robertsonian mutation indicated by arrow. **C.**, **D.** Metaphases from mutant *Sptbn1*^−/−^ MEFs. **C.** Metaphase showing robertsonian mutation indicated by (yellow arrow), fragment indicated by white arrow and end association indicated by blue arrow. **D.** Metaphase showing radial indicated by red arrow and fragment with blue arrow. **E.** Comparison of different types of chromosome aberrations in *Sptbn1*^−/−^ and *Sptbn1*^+/+^ MEFs. **F.** Western blot showing depletion of β2SP by specific siRNA and shRNA in human cells. **G.** Human metaphase chromosomes showing centromeres (green) and loss of telomeres (red) signals detected by FISH using specific probes. **H.** Metaphase from β2SP depleted cells showing dicentrics indicated by white arrow and telomere signals indicated by green arrow. **I.** Comparison of different types of aberrations seen in cells with and without depletion of β2SP. Quantification of aberrations from 300 metaphase spreads of three independent experiments. * *p* < 0.05; ** *p* < 0.01; ***p < 0.001 as determined by Student *t*- test.

We examined first whether β2SP-deficient cells exhibit spontaneous genomic instability by measuring chromosomal aberrations at metaphase in *Sptbn1*^−/−^ and *Sptbn1*^+/+^ MEFs. *Sptbn1^−/−^* MEFs had a significantly higher frequency of chromosomal aberrations of various types (fragments, radials and translocations like Robertsonian mutations) than wild-type MEFs (Figures 1A-1E). Furthermore, when SPTBN1 was depleted by specific siRNA in human cells (Figure 1F), a subsequent higher frequency of fragments, radials and dicentrics was observed (Figures 1G-1I). To determine whether telomere stability is affected by β2SP depletion, fluorescent *in situ* hybridization using telomere and centromere specific probes was used as described previous [19, 20]. Human β2SP deficient cells showed frequent loss of telomeres, which could result in telomere fusions that produce dicentrics, which were seen at a frequency similar to the translocations seen in mouse cells.

### Role of β2SP in cell survival after exposure to genotoxic agents

**Figure 2:**
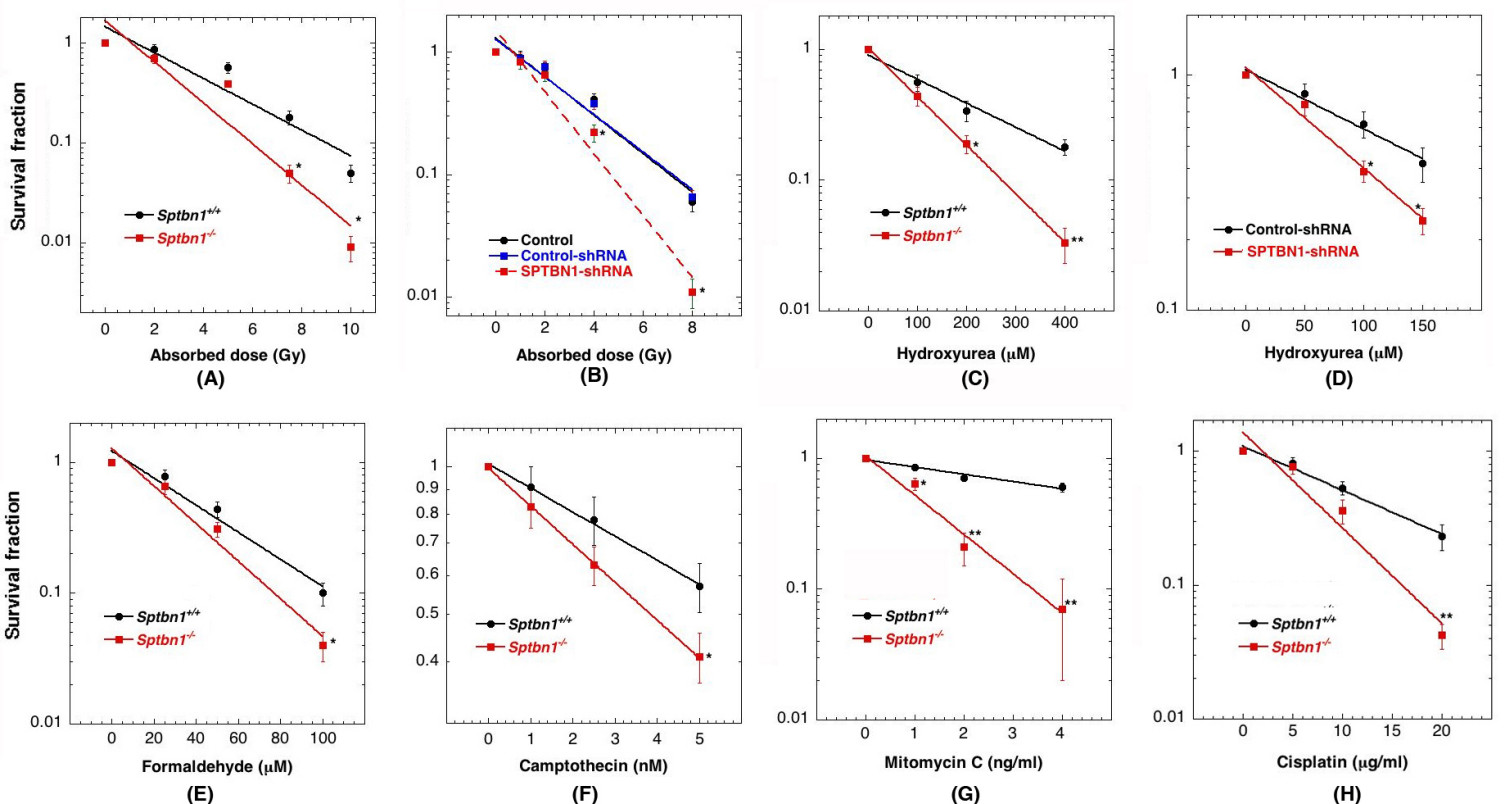
Cells depleted for β2SP have reduced cell survival after exposure to different genotoxic agents **A.** MEFs exposed to graded doses of IR at the dose rate of 1 Gy per min; **B.** Human cells with and without depletion of β2SP exposed to IR; **C.** MEFs treated with hydroxyurea; **D.** Human cells with and without depletion of β2SP treated with hydroxyurea; **E.** MEFs treated with formaldehyde; **F.** MEFs treated with camptothecin; **G.** MEFs treated with mitomycin C and **H.** MEFs treated with cisplatin.

We measured cell survival by assays described previously [21-24] and observed that exponentially growing *Sptbn1*^−/−^ MEFs and β2SP depleted human cells had a modest but statistically significant reduction in survival post irradiation as compared to the respective control cells (Figures 2A, 2B). Similarly, *Sptbn1*^−/−^ MEFs as well as β2SP depleted human cells were much more sensitive to hydroxyurea (HU) induced replication stress than control cells (Figures 2C, 2D). Since we observed that depletion of β2SP in mouse and human cells had similar effects on IR or HU induced cell killing, we then examined cell survival after treatment with formaldehyde, camptothecin, mitomycin C and cisplatin but only in MEFs (Figures 2E-2H) [25]. *Sptbn1*^−/−^ MEFs were more sensitive to intra- and interstrand cross-linking agents like formaldehyde, mitomycin C (MMC) and cisplatin as well as the topoisomerase inhibitor like camptothecin (Figures 2E-2H). All these agents reduced cell survival in *Sptbn1*^−/−^ MEFs as compared to *Sptbn1*^+/+^ MEFs, suggesting a role for β2SP in cell survival following exposure to genotoxic agents. Since cell killing usually correlates with defective DNA damage repair, we determined whether the reduced cell survival observed in β2SP depleted cells was the result of impaired DNA damage repair.

### β2SP-deficiency impairs DNA damage response

**Figure 3:**
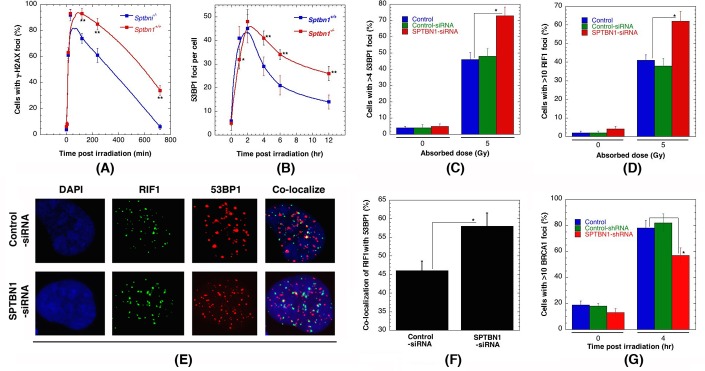
β2SP depletion impairs IR-induced repairsomes formation **A.** *Sptbn1*^−/−^ and *Sptbn1*^+/+^ MEFs were irradiated with 2 Gy then examined for γ-H2AX foci formation at different time points post irradiation. **B.** *Sptbn1*^−/−^ and *Sptbn1*^+/+^ MEFs were irradiated with 4 Gy examined for 53BP1 foci at different time points post irradiation. **C.** Quantification of human cells with 53BP1 foci, with and without β2SP depletion, 4 hour after exposure to 5 Gy. **D.** Quantification of human cells with RIFI foci, with and without β2SP depletion 4 hour after exposure to 5 Gy. **E.** Colocalization of RIF1 and 53BP1 foci in human cells with and without β2SP depletion. **F.** Quantification of colocalization of RIF1 and 53BP1 foci. **G**. Error bars represent standard deviations, 3 independent experiments were performed. *p < 0.05 and *p < 0.01 Student *t*-test.

One of the earliest detectable steps in the recruitment of repair factors to sites of DNA damage, particularly IR-induced DNA double-stranded breaks (DSBs), is phosphorylation of H2AX on serine 139 (γ-H2AX), which is linked to the activation of most DNA repair pathways [26-29]. Therefore, we initiallyasked if the IR-induced γ-H2AX response was perturbed by β2SP depletion. Upon exposure to IR, we found that γ-H2AX was equivalently detectable by 10 min in both of *Sptbn1*^−/−^ and *Sptbn1^+/+^* MEFs (Figure 3A). However, *Sptbn1*^−/−^ cells had markedly higher levels of residual γ-H2AX at later time points, suggesting abbreviated DNA damage processing compared to that of *Sptbn1^+/+^* MEFs (Figure 3A). This phenotype was recapitulated in human cells with β2SP depletion after 2 Gy IR exposure (data not shown). The initial appearance of 53BP1 foci (Figure 3B) in *Sptbn1*^−/−^ and *Sptbn1^+/+^* MEFs was also similar, however there was higher residual 53BP1 foci in *Sptbn1*^−/−^ MEFs as compared to *Sptbn1^+/+^* MEFs indicating defective recruitment of other factors involved in DNA damage repair. A higher level of residual 53BP1 foci was also seen in β2SP depleted human cells (Figure 3C), supporting the argument that the effect of β2SP on the DNA damage response is conserved in human and mouse cells. Since 53BP1 protein has been implicated in the regulation of DNA DSB pathway choice [30-32], and the first effector of 53BP1 is RIF1 [32-37], we compared the kinetics of IR-induced RIF1 foci appearance and disappearance in *Sptbn1*^−/−^ and *Sptbn1^+/+^* MEFs. Similar to 53BP1 status in β2SP depleted cells, higher residual RIF1 foci were observed in *Sptbn1*^−/−^ MEFs as compared to *Sptbn1^+/+^* MEFs, suggesting further that recruitment of repair associated proteins is effected by β2SP depletion (Figure 3C, 3D). *Sptbn1*^−/−^ MEFs also have a higher frequency of co-localized 53BP1 and RIF1 foci as compared to *Sptbn1^+/+^* MEFs (Figures 3E, 3F). Since the 53BP1-interacting protein RIF1 is critical for inhibition of DNA end resection in BRCA1-deficient cells [38], we compared IR-induced BRCA1 foci formation in *Sptbn1*^−/−^ and *Sptbn1^+/+^* MEFs and observed that β2SP loss reduced BRCA1 foci (Figure 3G), suggesting that β2SP promote recruitment of the resection machinery involved in HR-dependent repair.

**Figure 4:**
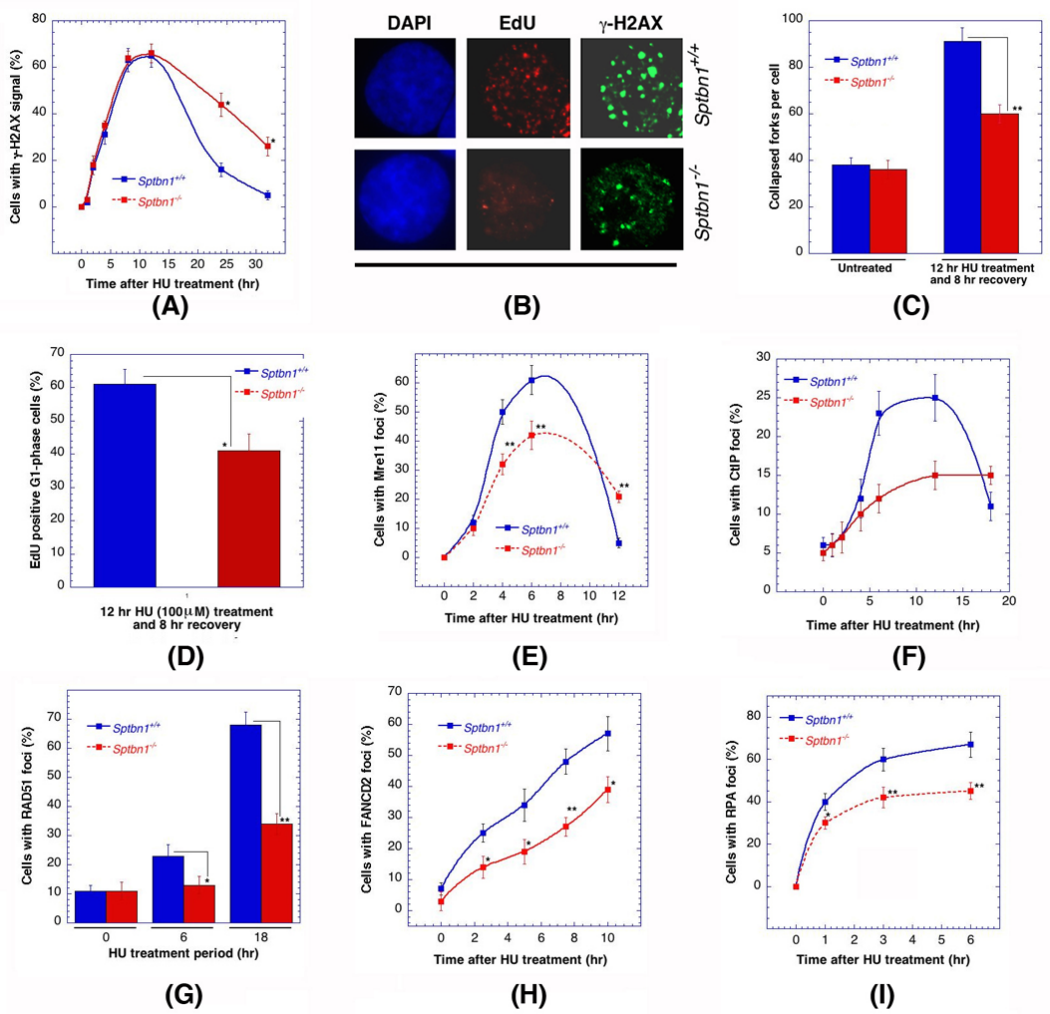
Comparison of *Sptbn1*−/− and *Sptbn1*+/+ MEFs for DNA damage response after hydroxyurea treatment **A.** Quantification of cells with γ-H2AX foci. **B.** Cells stained for DNA with DAPI and γ-H2AX immunostaining. EdU-positive S/G2 cells (selected by DAPI staining) were selected only for γ-H2AX focus counting. **C.** The number of larger γ-H2AX foci counted in late S/G2-phase cells, representing collapsed forks. The quantification of EdU-positive late S/G2-phase cells was performed as described previously [25]. **D.** Histogram showing percent EdU-positive G1 phase cells. A total of 3,000 cells were counted, and the means from three experiments are plotted. **E.**-**I.** Frequency of cells with Mre11 foci **E.**, CtIP foci **F.**, RAD51 foci **G.**, FANCD2 foci **H.** and RPA foci **I.**. *p < 0.05 and **p < 0.01, Student *t*-test.

Hydroxyurea treatment induces replicative stress and when exponential-phase cells with and without β2SP depletion were treated with the drug, a significant decrease in the disappearance kinetics of γ-H2AX foci was detected (Figure 4A) [39] as well as a reduced number of large γ-H2AX foci (Figure 4B), which are an indication of collapsed replication forks (Figure 4C). Delayed cell cycle progression was also observed as the frequency of G1 phase cells was significantly lower in cells depleted for β2SP (Figure 4D). Consistent with the abnormally sized γ-H2AX foci, a significant reduction in Mre11, CtIP, RAD51, FNACD2 and RPA foci formation was observed in hydroxyurea treated β2SP-deficient cells (Figures 4E-4I), Thus, the survival results, the defective disappearance kinetics of γ-H2AX foci and the reduced number of MRE11, CtIP, RAD51, FNACD2, and RPA foci after hydroxyurea treatment, all suggest that DNA damage repair is impaired in β2SP deficient cells and that β2SP may have a specific role in homologous recombination repair.

### Role of β2SP in S-phase specific chromosome damage repair

Cells depleted for β2SP have higher residual γ-H2AX and 53BP1 foci, and lower Rad51 and BRCA1 foci, indicating defective repair of DNA DSBs by the HR pathway, the repair pathway most commonly up regulated in S-phase cells. In addition, we found that *Sptbn1*^−/−^ MEFs have a higher frequency of 53BP1 and RIF1 foci co-localization than in *Sptbn1*^+/+^ MEFs even after 12 hr of HU treatment, suggesting that HR repair protein loading at DNA damage sites is impaired, an observation further supported by the reduced frequency of Rad51, BRAC1 foci in post treatment *Sptbn1*^−/−^ MEFs. To examine the cell cycle stage specific repair of IR-induced DNA damage, we examined chromosome aberrations produced at different phases of the cell cycle and detected in metaphase cells as described previously [20, 40, 41].

**Figure 5:**
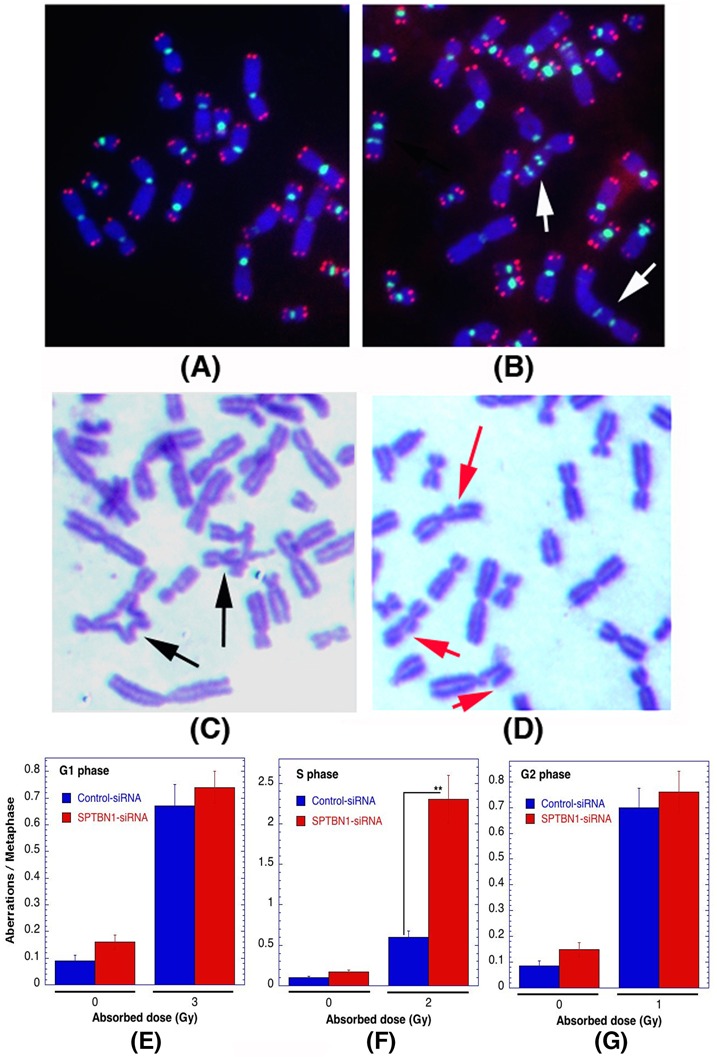
Cells deficient for β2SP show defective S-phase specific chromosome repair Cell cycle phase specific chromosomal aberrations induced by IR in human cells. **A.**, **B.** Metaphases of human cells showing centromeres green and telomeres red detected by FISH as described previously [19, 20, 63]. Metaphase chromosomes from control **A.** and G1-type metaphase chromosomes, arrows indicate dicentrics **B.**. **C.,D.** S- and G2-type chromosome aberrations detected by Giemsa staining as described previously [64, 65], S-phase **C.** showing radials indicated by black arrow **C.** and G2-phase **D.** showing breaks and gaps indicated by red arrows. **E.**-**G.** Quantification of chromosomal aberrations: G1-phase type **E.**, S-phase type **F.** and G2-phase type **G.**. **p < 0.01, Student *t*-test.

Cell cycle phase-specific chromosome aberrations were ascertained based on the frequency of chromosomal and chromatid-type aberrations observed at metaphase. G1-specific aberrations detected at metaphase are mostly of the chromosomal type and include a high frequency of dicentrics (Figures 5A, 5B) [42]. S-phase-type aberrations detected at metaphase are chromosomal, as well as those involving chromatids (Figure 5C). G2-type aberrations detected at metaphase are predominantly the chromatid type with the least number of dicentrics (Figure 5D). To determine G1-type chromosome damage, cells were treated with 3 Gy of IR and aberrations were scored at metaphase as previously described [40, 42]. No differences in residual IR-induced G1 chromosomal aberrations were detected in metaphase cells with or without β2SP depletion (Figure 5E). To determine whether defective repair can be documented in cells depleted for β2SP in other phases of the cell cycle other than G1, S-phase-specific chromosome aberrations were evaluated. Thus, cells were exposed with 2 Gy of IR and metaphases collected 4 to 6 h post irradiation. Cells depleted for β2SP collected post irradiation displayed higher frequencies of metaphases with chromatid and chromosomal aberrations than control cells (Figure 5F). In contrast, when cells were treated with 1 Gy of IR, depletion of β2SP had minimum effect on G2-phase-specific chromosome repair (Figure 5G). These observations suggest a role for β2SP in repairing chromosome damage in the S phase of the cell cycle, where HR is up-regulated.

### β2SP-deficient cells are defective in resolution of stalled DNA replication forks

**Figure 6:**
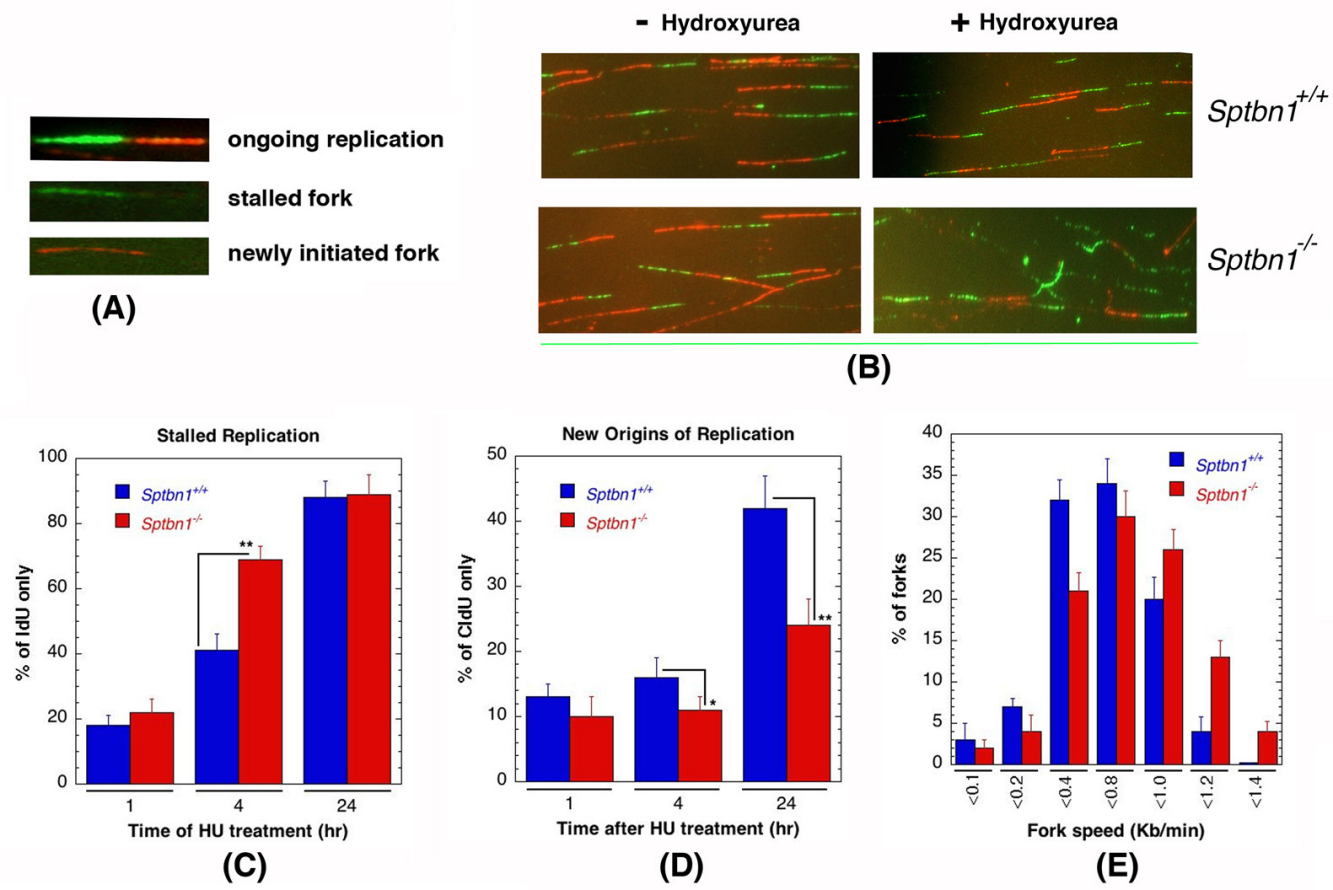
*Sptbn1*−/− MEFs exhibit defective stalled DNA replication fork resolution and new origins of replication **A.**, **B.** Initiation of new DNA replication forks and reinitiation of stalled DNA replication forks in *Sptbn1*^−/−^ and *Sptbn1*^+/+^ MEFs. cells were pre-labeled with 5-iododeoxyuridine (IdU), treated with hydroxyurea (HU) for the indicated intervals, and then rinsed to remove HU followed by post labeling with 5-chlorodeoxyuridine (CldU) (upper panel) as described previously [66]. The cells were fixed and immunostained with IdU (green) and CldU (red) antibodies. **A.** Three major types of labeled DNA tracts for analysis are shown. **B.** *Sptbn1*^+/+^ and *Sptbn1*^−/−^ MEFs with and without treatment of hydroxyurea. **C.** Percentages of stalled DNA replication forks (IdU only signals) in *Sptbn1*^−/−^ and *Sptbn1*^+/+^ cells after HU treatment. **D.** New origins (CldU signals) in *Sptbn1*^−/−^ and *Sptbn1*^+/+^ cells. **E.** Percentage of forks with fork speed in *Sptbn1*^−/−^ and *Sptbn1*^+/+^ MEFs. Means ± standard deviations of 3 independent experiments are shown in. *p < 0.05; **p < 0.01, Student *t*-test.

The disappearance of γ-H2AX foci in β2SP deficient cells treated with HU was significantly delayed (Figure 4A) and they also had a reduced number of large γ-H2AX foci (Figure 4B) as compared to controls. *Sptbn1*^−/−^ cells treated with HU have a high frequency of collapsed forks (Figure 4C) and cell cycle progression is delayed as the frequency of G1 phase cells was significantly lower (Figure 4D). To determine the mechanism by which *Sptbn1* deficiency impacts the resolution of stalled DNA replication forks after DNA repair, we compared the restart of stalled forks in cells with and without *Sptbn1* using the DNA fiber technique [25, 43]. Mouse embryonic fibroblasts were pulse-labeled with 5-iododeoxyuridine (IdU), treated with HU for different time periods to deplete the nucleotide pool, and then washed and pulse-labeled with 5-chlorodeoxyuridine (CldU) (Figures 6A, 6B) as described previously [25, 43]. In growing cells continuously exposed to HU, we observed a higher percentage of stalled DNA replication forks (IdU signals) in mutant *Sptbn1*^−/−^ cells than in wild type *Sptbn1*^+/+^ cells after 4 hours of treatment (Figure 6C). Moreover, after removal of the HU block, we observed fewer contiguous IdU/CldU signals in *Sptbn1*^−/−^ cells, indicating that the restart of previously initiated replication origins in the β2SP deficient cells was decreased as compared to control cells, which readily resumed DNA synthesis (Figure 6D). The deficiency of β2SP appeared to decrease fork speed as *Sptbn1*^−/−^ MEFs had a relatively low frequency of fibers with large labeled fork fragments as compared to *Sptbn1*^+/+^ MEFs (Figure 6E).

### β2SP depletion affects DNA resection

**Figure 7:**
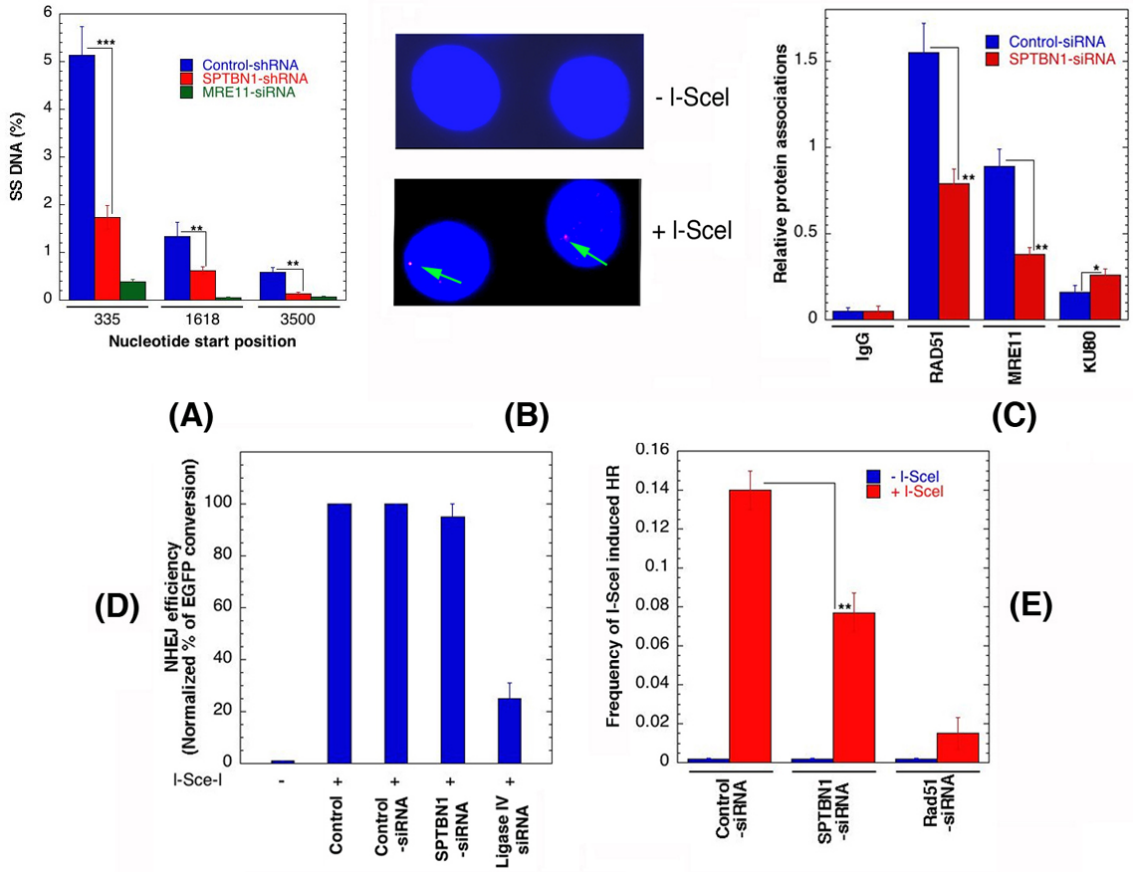
β2SP depletion affects DNA resection and DSB repair by homologous recombination **A.** Quantification of single strand DNA (ssDNA) in cells with and without depletion of β2SP. **B.** Detection of I-SceI induced DSB by γ-H2AX immunostaining. Note arrow indicates the site of γ-H2AX foci in cells expressing I-SceI. **C.** Detection of repair proteins at l-Scel sites by ChIP using the respective antibodies. **D.** Non-homologous end joining frequencies in human cells are shown after I-SceI induction in cells with and without depletion of β2SP. **E.** HR frequencies in human cells are shown with and without I-SceI induction. **p < 0.01, Student *t*-test.

A resection assay was used to quantitate ssDNA at DSBs, induced in human cells by AsiSI endonuclease in the presence and absence of β2SP. The AsiSI enzyme, fused to the estrogen receptor hormone-binding domain, translocates to the nucleus following 4-OHT treatment to generate sequence-specific DSBs (5′-GCGATCGC-3′) [45]. To measure resection adjacent to specific DSBs, a AsiSI site known to be cleaved with high efficiency on Chromosome 1 (DSB1, Chr 1: 88992915 [GRCh38/hg38]) was examined. Three pairs of qPCR primers were used that amplify across BsrGI restriction sites at various distances from each AsiSI site [46]. The percentage DSBs resected was measured by (% ssDNA) / (% DSB) at the sites after 4 hr of 4-OHT treatment. Depletion of β2SP decreased resection both immediately adjacent to the DSB site as well as further downstream of the site (Figure 7A). This result was similar to that observed in MRE11 depleted cells, a factor known to be required for DNA end resection during HR repair.

The frequency of RIF1 colocalization with 53BP1 is significantly higher in post irradiated human β2SP depleted cells than in control cells. Consistent with results that β2SP has a role in HR, there was a reduced frequency of cells with IR-induced BRCA1, CtIP, RAD51, RPA2, MRE11, RAP80, and FANCD2 foci in β2SP depleted cells, supporting a direct link to DSB repair by HR. To further examine the role of β2SP in DNA repair protein recruitment upon DSB induction, we determined the levels of RAD51, MRE11 and KU80 at the DSB site before and after DSB induction in cells with and without β2SP depletion (Figure 7B). We compared the levels of RAD51, MRE11 and KU80 at different distances from an I-SceI induced DSB site using ChIP analysis with site specific primers [47]. The HR related proteins Rad51 and MRE11 accumulated at the I-SceI induced DSB site to relatively lower levels in β2SP depleted cells, where as KU80 levels were increased by depletion (Figure 7C). Since β2SP depletion reduces the formation of IR-induced RAD51 foci and RAD51 localization at a defined DSB site, a hallmark of HR [27, 48], we examined individually the effect of β2SP depletion on non-homologous end joining (NHEJ) and HR based I-SceI induced DSB repair.

### β2SP depletion affects DNA DSB repair by homologous recombination

Depletion of β2SP affects both the DNA resection process as well as recruitment of DNA repair proteins, therefore, we assessed the impact of β2SP-deficiency on the two key DNA damage repair pathways: HR and NHEJ. We exploited *in vivo* reporter systems for either HR or NHEJ in order to study the impact of β2SP knockdowns on DSB repair [23, 44] by using engineered cell lines that express a fluorescent protein only upon repair of I-SceI induced DSBs by the appropriate pathway [23, 33, 48]. Equivalent repair of the NHEJ-dependent fluorescent protein gene template was observed in cells with and without depletion of β2SP (Figure 7D), further confirming that β2SP is not required for DSB repair by the NHEJ pathway. This is also consistent with the normal chromosomal damage repair observed in β2SP depleted G1 phase cells. The reconstitution frequency of a GFP HR reporter gene within a chromosomally integrated plasmid substrate [20, 49] was used to measure the dependence of HR on β2SP. In cells depleted of β2SP, the HR repair was defective as the number of GFP-positive cells was significantly decreased as compared to control (Figure 7E). These data suggest that β2SP is required for DNA DSB repair by the HR pathway but not by NHEJ. Overall, these results indicate that β2SP loss results in DNA damage repair defects at the level of stalled replication fork resolution and HR, leading to chromosome aberrations in an S-phase specific manner.

## DISCUSSION

SPTBN1 has emerged as a potent regulator of tumorigenesis as mice haploinsufficient for β2SP (*Sptbn1^+/−^*) spontaneously develop hepatocellular carcinoma HCC [12]. Our study now uncovers a novel role for β2SP in DNA damage repair and maintenance of genomic stability. Thus, β2SP signaling is pivotal not only for regulation of hepatocyte proliferation and extracellular matrix deposition in the liver, as previously reported [50-53], but also for maintenance of genomic stability in the context of toxin-induced DNA damage. Indeed, TGF-β is activated in response to tissue damage [54]. Interestingly, only double knockout *Fancd2/Aldh2* mice display fetal alcohol syndrome (FAS), indicating that elevated genotoxic acetaldehyde was required for the FAS phenotype. When there is a deficiency of β2SP, it is possible that the TGF-β pathway has additional sensitizing effects, thus rendering the mice more susceptible to DNA damage. For instance, TGF-β plays a role in the reciprocal up-regulation of TLR4 signaling [55].

It is well established that 53BP1 enhances DSB repair through the error-prone NHEJ pathway by blocking the recruitment of resection proteins associated with HR repair [38, 56, 57]. In our analysis, cells with β2SP depletion had higher levels of residual 53BP1 foci as well as increased co-localization of 53BP1 and RIF1 supporting the argument β2SP plays a critical role in regulating the DNA DSB repair, specifically by the HR pathway. In addition DNA replication fork stalling and reduced initiation of new DNA replication forks are the likely underlying mechanistic basis for defective DNA interstrand cross-link repair observed in *Sptbn1-*deficient cells.

Recent studies by Zhi and coworkers also demonstrated that *Sptbn1* gene expression is positively correlated with E-cadherin and kallistatin expression in both HCV-associated HCC (Gene Expression Omnibus GSE6764) and hepatitis B virus (HBV)-associated HCC (Gene Expression Omnibus GSE14520) [58]. These data suggest that β2SP plays a tumor suppressive role through different signaling pathways in HCC. It is possible that the TGF-β/β2SP/SMAD3/FANCD2 axis may also be activated in response to virus-induced DNA damage and genomic instability, but this remains to be investigated further.

The present results validate recent discoveries that cytoskeletal proteins belonging to the spectrin/ankyrin family can regulate key signal transduction pathways by functioning as adaptor molecules [13]. Moreover, our results reinforce the concept that consideration of the role of these proteins in human physiology and disease may be pivotal for the development of new therapeutics acting through DNA damage repair pathways that aim to change the diseased state from injury to repair. The complex detoxifying function of the liver clearly exposes the organ to a phenomenal degree of genotoxic stress, which must be overcome in order to prevent DNA damage and the potential onset of liver cirrhosis and cancer. Our studies propose that in response to toxins, β2SP directly affects DNA repair to maintain genomic stability. Thus, characterizing the role of TGF-β in alcohol-induced injury could potentially enhance our mechanistic insight into the basis for therapeutics targeting reactive aldehydes and DNA repair.

## MATERIALS AND METHODS

### Cell culture, transfection and cell survival

Human liver cancer cell line HepG2 (HB8065; ATCC, Rockville, MD) was cultured in DME/F12 medium (D5671; Sigma Aldrich, St Louis, MO) supplemented with 10% fetal bovine serum (F2442; Sigma Aldrich). For transfection, Lipofectamine 2000 (Invitrogen, Carlsbad, CA) was used. shRNAs for β2SP (sc-36551-v) and shRNA control (sc-108080) were from Santa Cruz Biotechnology (Santa Cruz, CA), siRNAs were from Dharmacon (Lafayette, CO). For survival assay, three different assays were used: (a) regrowth curve, (b) microtiter well, and (c) clonogenic colony formation as described previously [21, 22]

### Immunostaining, foci measurements, NHEJ and HR assay

Cells were grown in chamber slides, fixed in 2% paraformaldehyde for 15 min, washed in phosphate-buffered saline (PBS), permeabilized for 5 min on ice in 0.2% Triton X-100, and blocked in PBS with 1% bovine serum albumin. The procedure used for immunostaining is the same as that described previously [33, 59, 60]. Foci were counted by microscopic and imaging processes. Fluorescence images were captured by using epifluorescence microscope equipped with a charge-coupled device camera and software. Optical sections through nuclei were captured, and the images were obtained by projection of the individual sections. The results shown are from three independent experiments. Cells with a bubble like appearance or micronuclei were not considered for foci analysis. Antibodies used have been described previously [25, 33, 61, 62]. The impact of β2SP on DNA DSB repair by NHEJ and HR was determined by using both IR- as well as I-Scel induced DNA DSB in fluorescent protein reconstitution assays. The impact of β2SP on repair protein recruitment to a defined I-SceI DSB was determined by ChIP and PCR. Induction of a site specific DSB was performed as described previously [23]. Commercial antibodies used have been described previously [23, 25, 61, 62]

### DNA replication restart assay

Mouse embryonic fibroblasts were pulse labeled with 50 μM IdU for 20 minutes, washed 3 times with 1X PBS, treated with 2 mM HU for the indicated intervals, washed 3 times with PBS to remove HU, and then incubated in fresh medium containing 50 μM CldU for 20 minutes, and then washed 3 times with PBS. DNA fiber spreads were made using a modified procedure described previously [25]. Briefly, cells labeled with IdU and CldU were mixed with unlabeled cells in a ratio of 1:10, and 2 μL cell suspensions were dropped onto a glass slide and mixed with a 20 μL hypotonic lysis solution for 8 minutes. Air-dried slides were fixed, and incubated with primary antibodies (rat monoclonal antibody anti-IdU [Abcam, Cambridge, MA] and mouse monoclonal antibody anti-CldU [BD Biosciences, San Jose, CA]) and secondary antibodies (anti-rat Alexa Fluor 488- conjugated and anti-mouse Alexa Fluor 568-conjugated antibodies). Image J software was used to analyze the DNA fibers.

### Chromosomal aberration analysis

For analysis of G1-phase-specific chromosome aberrations, cells were irradiated (3 Gy), incubated for 10-12 hours, and then treated for 3 hours with colcemid, followed by hypotonic treatment and fixation for scoring metaphase chromosome aberrations [20]. Categories of asymmetric chromosome aberrations scored included Robertsonian fusions, dicentrics, centric rings, interstitial deletions, acentric fragments, rings, and terminal deletions [23]. For S-phase-specific chromosome aberrations, cells were irradiated (2 Gy) and incubated for 4-6 hours, and cells at metaphase were harvested after 3 hours of treatment with colcemid. S-phase-specific aberrations observed in the first round of cells at metaphase included triradials and quadriradials, breaks, and gaps [33]. For G2-type chromosome aberrations, exponential-phase cells were irradiated (1 Gy), incubated for 1 hour, and treated with colcemid for 3 hours, followed by hypotonic treatment fixation to analyze chromosome aberrations at metaphases [23]. The analysis of metaphase spreads to measure chromosomal aberrations was carried out as described previously [20, 23].

### Statistical analysis

Differences between groups were evaluated using Student *t* tests. Results are expressed as mean ± standard deviation. For all statistical analyses, *p* < 0.05 was considered statistically significant.
