# Correction: β2-spectrin depletion impairs DNA damage repair

**DOI:** 10.18632/oncotarget.28857

**Published:** 2026-04-24

**Authors:** Nobuo Horikoshi, Raj K. Pandita, Kalpana Mujoo, Shashank Hambarde, Dharmendra Sharma, Abid R. Mattoo, Sharmistha Chakraborty, Vijaya Charaka, Clayton R. Hunt, Tej K. Pandita

**Affiliations:** ^1^Department of Radiation Oncology, Houston Methodist Research Institute, Houston, TX, USA; ^2^Department of Radiation Oncology, University of Texas Southwestern Medical School, Dallas, TX, USA; ^3^Department of Microbiology and Molecular Genetics, McGovern Medical School, University of Texas Health Science Center at Houston, Houston, TX, USA

**This article has been corrected:** It was identified that a portion of Figure 6B was improperly placed, resulting in an overlap between two images (*Sptbn1*^−/−^ and *Sptbn1*^+/+^) for new DNA replication forks in MEF cells without Hydroxyurea treatment. The authors provided a revised Figure 6B, replacing the image for *Sptbn1*^−/−^ cells without Hydroxyurea treatment with the correct data from the original experiments. This correction does not alter the article’s conclusions.

Original article: Oncotarget. 2016; 7:33557–33570. 33557-33570. https://doi.org/10.18632/oncotarget.9677

**Figure 6:**
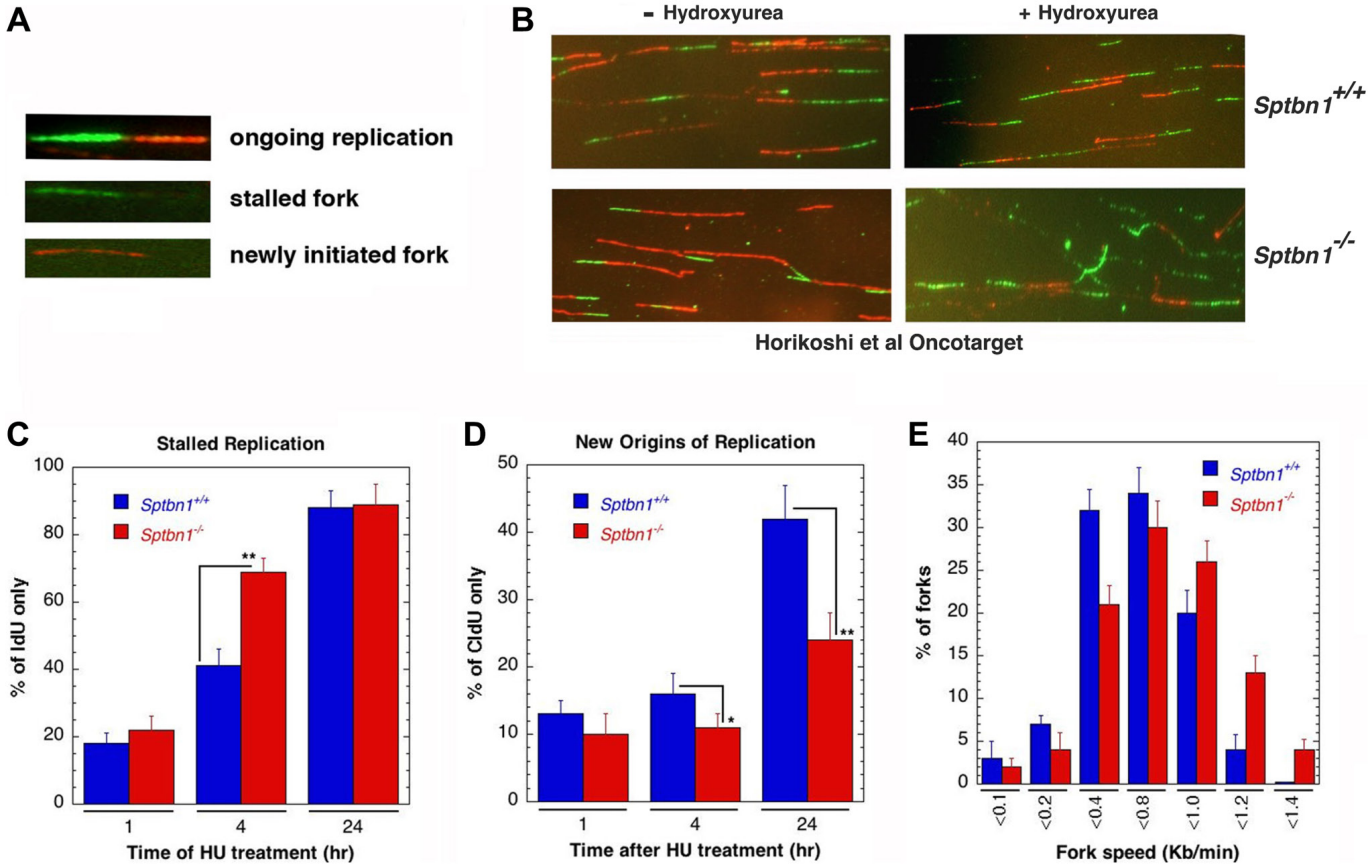
*Sptbn1*^−/−^ MEFs exhibit defective stalled DNA replication fork resolution and new origins of replication. (**A**, **B**) Initiation of new DNA replication forks and reinitiation of stalled DNA replication forks in *Sptbn1*^−/−^ and *Sptbn1*^+/+^ MEFs. cells were pre-labeled with 5-iododeoxyuridine (IdU), treated with hydroxyurea (HU) for the indicated intervals, and then rinsed to remove HU followed by post labeling with 5-chlorodeoxyuridine (CldU) (upper panel) as described previously [66]. The cells were fixed and immunostained with IdU (green) and CldU (red) antibodies. (A) Three major types of labeled DNA tracts for analysis are shown. (B) *Sptbn1*^+/+^ and *Sptbn1*^−/−^ MEFs with and without treatment of hydroxyurea. (**C**) Percentages of stalled DNA replication forks (IdU only signals) in *Sptbn1*^−/−^ and *Sptbn1*^+/+^ cells after HU treatment. (**D**) New origins (CldU signals) in *Sptbn1^−/−^* and *Sptbn1*^+/+^ cells. (**E**) Percentage of forks with fork speed in *Sptbn1^−/−^* and *Sptbn1*^+/+^ MEFs. Means ± standard deviations of 3 independent experiments are shown in. ^*^p < 0.05;^ **^p < 0.01, Student *t*-test.

